# *Cannabis* Medicine 2.0: Nanotechnology-Based Delivery Systems for Synthetic and Chemically Modified Cannabinoids for Enhanced Therapeutic Performance

**DOI:** 10.3390/nano15161260

**Published:** 2025-08-15

**Authors:** Izabela Żółnowska, Aleksandra Gostyńska-Stawna, Anna Jelińska, Maciej Stawny

**Affiliations:** 1 Department of Pharmaceutical Chemistry, Poznan University of Medical Sciences, Rokietnicka 3, 60-806 Poznan, Poland; ajelinsk@ump.edu.pl (A.J.); mstawny@ump.edu.pl (M.S.); 2 Doctoral School, Poznan University of Medical Sciences, Bukowska 70, 60-812 Poznan, Poland

**Keywords:** synthetic cannabinoids, nanotechnology, nanoformulations, cannabinoid structure

## Abstract

The therapeutic potential of cannabinoids and other ligands of cannabinoid receptors attracts considerable attention due to their diverse pharmacological effects and utility in various medical applications. However, challenges such as low solubility, limited bioavailability, and potential side effects hinder their broad clinical use. Nanoformulation techniques offer a promising approach to address these issues and optimize the therapeutic effectiveness of cannabinoids and other cannabinoid receptor ligands. This comprehensive review explores the advancements in nanoformulation strategies to enhance the therapeutic efficacy and safety of synthetic cannabinoids and related compounds, such as CB13, rimonabant, and HU-211, which have been studied in a range of preclinical models addressing conditions such as neuropathic pain, depression, and cancer. The review discusses various nanocarriers employed in this field, including lipid-based, polymeric, and hybrid nanoparticles, micelles, emulsions, and other nanoengineered carriers. In addition to formulation approaches, this review provides an in-depth analysis of chemical structures and their effect on compound activity, especially in the context of the affinity for the cannabinoid type 1 receptor in the brain, which is chiefly responsible for the psychoactive effects. The provided summary of research concerning either chemical modifications of existing cannabinoids or the creation of new compounds that interact with cannabinoid receptors, followed by the development of nanoformulations for these agents, allows for the identification of new research directions and future perspectives for *Cannabis*-based medicine. In conclusion, the combination of nanotechnology and cannabinoid pharmacology holds promise for delivering more effective and safer therapeutic solutions for a broad spectrum of medical conditions, making this an exciting area of research with profound implications for the healthcare and pharmaceutical industries.

## 1. Introduction

*Cannabis sativa* L., a plant species belonging to the *Cannabaceae* family [1], holds a long tradition of utilization by human societies [2]. Different strains of this herb have been employed in a multitude of ways. *Cannabis* seeds have served as a raw material for obtaining oil used for culinary purposes or as a component of cosmetics. The plant’s fibers have found applications in producing paper, building materials, and fabrics [3]. Moreover, *Cannabis* has a history of medical use, with formulations derived from the plant employed to treat various conditions, such as rheumatism, different types of pain, asthma, and sleep disorders, as well as to enhance cheerfulness and stimulate appetite in patients [4].

The understanding of the mechanism behind *Cannabis* activity was significantly improved with the discovery in the 1960s of the substances produced by the plant, named cannabinoids. Continued research over the next 30 years led to the identification of receptors in the body that serve as docking sites for these compounds [5]. So far, two cannabinoid receptors—CB1 and CB2—have been distinguished, both belonging to the family of G-protein coupled receptors. The CB1 receptor is primarily found in the brain. However, it is also expressed in the spleen, thymus, heart, lungs, and vasculature. In contrast to the CB1, the CB2 receptor is mainly located in peripheral tissues. It is present in leukocytes, the spleen, thymus, tonsils, lungs, and gonads, and it regulates the functioning of several systems: cardiovascular, respiratory, skeletal, digestive, reproductive, and nervous [6].

**Figure 1:**
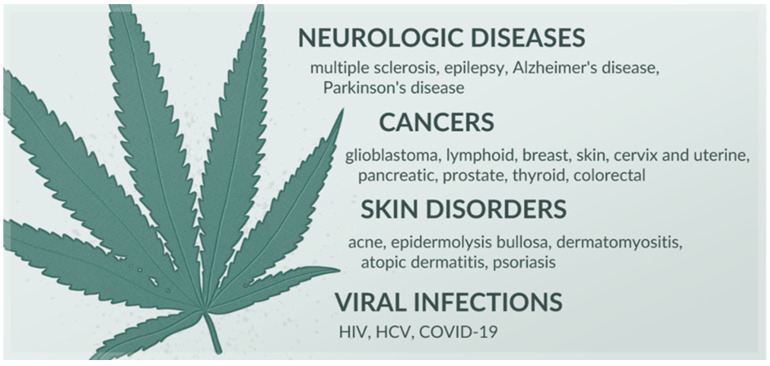
Diseases in which cannabinoids exhibit therapeutic potential, based on [8,9].

Among the over 100 cannabinoids isolated from *Cannabis*, Δ^9^-tetrahydrocannabinol (THC) and cannabidiol (CBD) are the most prominent [6]. Chemically, they share a characteristic C21 terpenophenolic backbone common to cannabinoids [7]. THC readily crosses the blood–brain barrier, stimulating the CB1 receptor and decreasing intracellular levels of cAMP. This mechanism underpins the well-known psychoactive effects of *Cannabis*. THC also possesses anti-inflammatory, antispasmodic, and neuroprotective activities. These are attributed to its action as a partial agonist of the CB2 receptor and its regulatory impact on non-cannabinoid receptors, such as peroxisome proliferator-activated receptor gamma (PPARγ), transient receptor potential vanilloid type 2 (TRPV2), type 3 (TRPV3), and type 4 (TRPV4). CBD does not demonstrate a psychoactive effect, unlike THC, and can even mitigate such an activity. It functions as an inverse agonist of the CB2 receptor and additionally interacts with serotonin-1A receptor (5HT1A), PPAR-γ, TRPV1, and TRPV2 receptors. CBD exhibits a range of effects, including anti-inflammatory, immunosuppressive, immunomodulatory, analgesic, anxiolytic, antioxidant, and anticonvulsant. Other phytocannabinoids, similar to THC and CBD, also demonstrate pleiotropic effects by modulating the functioning of various receptors and enzymes [8]. Due to their activity, cannabinoids hold promise in managing a variety of disorders, including neurologic illnesses [8,9], cancers, viral infections, and dermatological diseases (Figure 1) [8]. In certain conditions, cannabinoid-containing medicinal products are already utilized in healthcare—one such formulation approved by the European Medicines Agency is Epidyolex. This is an oral solution containing 10% CBD, indicated for intractable childhood epilepsy [10].

Despite the plethora of benefits arising from its use, *Cannabis* is not extensively exploited in medicine, partially due to its psychoactive activity [11]. Euphoria, dizziness, nausea, disorientation, and anxiety—the side effects primarily induced by THC—differ greatly in their severity among participants in conducted studies. This variability can be attributed to differences in study methodologies, including the use of diverse cannabinoid ratios and doses. Furthermore, distinct pharmaceutical formulations were applied in the research, potentially influencing the observed results [12]. Cannabinoids are highly lipophilic compounds, negatively impacting their solubility in water and posing a challenge in developing optimal formulations that can effectively deliver the drug to the body [13]. This issue is prevalent with many naturally derived substances [14,15,16,17,18]. Multiple dosage forms of cannabinoids, both oral and inhaled, are already under investigation. It is well known that the route of administration affects the pharmacokinetics of the active substance. Orally consumed cannabinoids have a longer duration of action, but the onset is relatively late. This stems, among other aspects, from the absorption rate, which may be affected by numerous factors. Conversely, inhaled cannabinoids act faster, with a shorter duration but greater potency, increasing the likelihood of side effects. The discrepancies in bioavailability resulting from utilizing different formulations complicate the interpretation of research outcomes related to the effects of cannabinoids. Differences between cannabinoid products also arise from the plants’ quality [12], as *Cannabis* can grow in different climates and be harvested under different conditions [11]. Adding to all these issues is the matter of using plant extracts without investigating their composition, where, apart from cannabinoids, the extracts also contain other groups of biologically active compounds, including flavonoids and terpenes. In such cases, it becomes impossible to determine which compound and at what dose truly caused the observed effect [19]. This lack of standardization has resulted in a limited number of high-quality clinical studies conducted so far, which consequently restricts the widespread use of *Cannabis* in medicine [12].

Various approaches are being explored to overcome the limitations associated with *Cannabis* treatment. This article offers an overview of research concerning a two-step strategy for enhancing cannabinoid system-related therapies. The first step involves the derivatization of known cannabinoids or the development of novel ligands targeting cannabinoid receptors, aiming to improve bioavailability and reduce side effects. The second step focuses on the application of nanoformulation techniques to these modified or synthetic compounds to further enhance therapeutic potential and optimize drug delivery.

Nanoformulation strategies encompass a wide variety of nanocarriers that can be classified based on their structural and material composition. These include lipid-based carriers, such as solid lipid nanoparticles (SLNs), nanostructured lipid carriers (NLCs), liposomes, and nanoemulsions (NEs); polymeric carriers, including poly(lactic-co-glycolic acid) (PLGA), polycaprolactone (PCL), polyethylene glycol (PEG), and polymeric micelles; and inorganic and hybrid systems, such as mesoporous silica, metallic nanoparticles, dendrimers, and cyclodextrin inclusion complexes. Each of these classes offers distinct advantages in terms of drug encapsulation efficiency, release kinetics, targeting capabilities, and biocompatibility. In cannabinoid delivery, lipid-based and polymer-based systems have shown particular promise for enhancing solubility, stability, and site-specific activity of active compounds [20,21,22,23].

## 2. Modification of Cannabinoid Structure

As the properties of cannabinoids are influenced by their molecular characteristics, various chemical modifications have been explored to improve their pharmacological profile. Cannabinoids, defined as compounds capable of binding to CB1 or CB2 receptors, regardless of their chemical structure, are categorized into three main types: phytocannabinoids, endocannabinoids, and synthetic cannabinoids [24,25]. Each group exhibits distinct molecular properties and elicits a range of biological effects. Phytocannabinoids are structurally based on 2-substituted 5-amylresorcinol and are characterized by a benzopyran ring with varying aliphatic side chains. For instance, THC’s pentyl side chain enhances its binding affinity for CB1 receptors, contributing to its psychoactive effects. Structural differences in the side chains and substitutions among phytocannabinoids result in varied receptor affinities and biological activities [24,25,26].

Endocannabinoids are endogenous ligands, typically derived from arachidonic acid, which interact with cannabinoid receptors to maintain physiological homeostasis. Anandamide (AEA) and 2-arachidonoylglycerol (2-AG) are the primary endocannabinoids, containing long-chain unsaturated fatty acids linked to ethanolamine (in AEA) via an amide bond or to glycerol (in 2-AG) via an ester bond [27]. These structural features enable selective receptor binding, impacting processes such as pain modulation, immune response, and mood regulation. Endocannabinoids are not stored in tissues but are synthesized only as needed, primarily by neurons and glial cells in the central nervous system (CNS), as well as by diverse cell types, such as immune cells, adipocytes, smooth muscle cells, endothelial cells, and fibroblasts [5,28]. This responsive synthesis enables rapid modulation of the body’s reaction to physiological stimuli such as pain, stress, and inflammation, allowing for precise regulation of functions within the endocannabinoid system [5,27].

Synthetic cannabinoids are laboratory-designed compounds created to mimic or enhance cannabinoid effects, often with structural modifications to avoid legal restrictions. These cannabinoids belong to several chemical classes, such as naphthoylindoles, cyclohexylphenols, and aminoalkylindoles. Modifications such as cyclopropyl or adamantyl groups enhance binding to CB1 or CB2 receptors, frequently leading to more intense psychoactive effects. For example, JWH-018, a naphthoylindole, binds strongly to CB1 receptors, producing potent psychoactivity [29].

**Figure 2:**
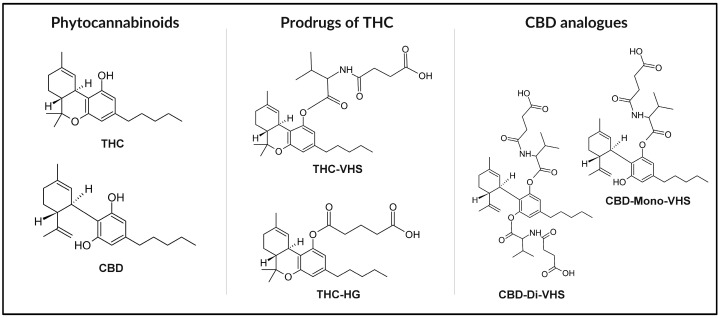
Chemical structures of THC and CBD with selected derivatives: THC-VHS, THC-HG, CBD-Mono-VHS, and CBD-Di-VHS.

Natural and synthetic cannabinoids exhibit low water solubility, limiting their potential for disease prevention and treatment [30]. One strategy to enhance cannabinoid solubility involves modifying their chemical structure. This approach can lead to the development of prodrugs, initially inactive compounds that become therapeutically active upon biotransformation within the body [31]. Prodrug synthesis is a known method in drug development, with approximately 10% of available market drugs existing in such a form [32]. Derivatized cannabinoids that are not prodrugs can also find applications in the therapy of various diseases due to their biological activity. In addition to modifying the structure of known cannabinoids, de novo synthesis of ligands for cannabinoid receptors is also possible [13]. Compounds have been developed that do not penetrate the blood–brain barrier, thereby preventing the psychoactive effects associated with Cannabis use [33]. Apart from improved water solubility, synthetic compounds offer additional advantages, such as consistency in composition due to the absence of standardization issues. Unlike the use of the plant, their application yields reproducible effects. However, it should be noted that employing specific isolated cannabinoids eliminates the synergistic effects of multiple compounds observed with the administration of whole-plant extracts [19]. The structures of THC and CBD, along with their selected chemical derivatives (Δ^9^-tetrahydrocannabinol-valine-hemisuccinate (THC-VHS), hemiglutarate ester prodrug of THC (THC-HG), CBD–monovalinate–monohemisuccinate (CBD-mono-VHS), and CBD-divalinate-dihemisuccinate (CBD-di-VHS)) designed to improve physicochemical properties, are presented in Figure 2.

## 3. Cannabinoids’ Nanoformulations

Beyond structural optimization, efforts have also focused on improving the delivery of cannabinoids through formulation strategies. Many researchers focus on the development of appropriate cannabinoid drug delivery systems. THC, due to its hydrophobic nature, has shown increased bioavailability when dissolved in oil and administered orally compared to an extract, although it only increased from 6% to 10–20%. However, from a practical standpoint, consuming undiluted oils is unpleasant [13]. Orally administered CBD has a bioavailability of only 9–13% due to its poor solubility and extensive first-pass hepatic metabolism [31]. Therefore, there is a need for formulations that effectively deliver cannabinoids to the body [13]. In recent years, diverse nanotechnology-based systems have gained increasing interest in this context [34]. Various formulations have been tested, including microparticles, emulsions, hydrogels, and emulgels [35,36]. They not only provide enhanced solubility of encapsulated cannabinoids but also improve their stability and enable site-specific delivery [34].

**Figure 3:**
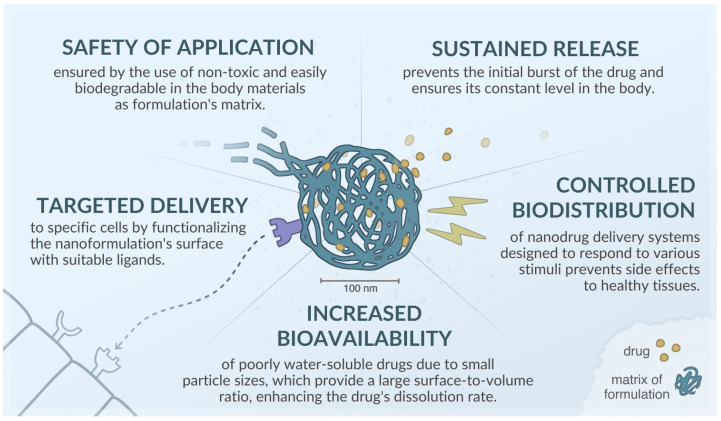
Advantages of nanoformulations, based on [40,41,42,43].

Key advantages of drug carriers at the nanometer scale include their small particle size, which is crucial, as the effectiveness of numerous medicines depends on this factor. The reduced particle size, accompanied by an increased contact surface area, generally facilitates efficient absorption by various cells, enhancing solubility and, as a result, improving the bioavailability of the drug [37]. Moreover, nanocarriers can penetrate the tumor endothelium or be absorbed by the tight junctions of skin endothelial cells. In intravenous administration, smaller sizes are also preferred due to a lower risk of embolism [38,39]. A summary of the advantages of using nanocarriers for active substances is presented in Figure 3.

Attempts to incorporate phytocannabinoids into various nanoformulations have been described in the literature, e.g., THC has been encapsulated in nanoparticles (NPs) [44,45] and NLCs [46]; CBD has been incorporated into NPs [47,48,49,50,51,52], micelles (MCs) [53,54], NEs [55,56,57,58], and self-nanoemulsifying drug delivery systems (SNEDDS) [59,60,61]; and a combination of THC and CBD has been encapsulated in pro-nanolipospheres [62,63] and SNEDDS [64,65]. Review articles summarizing nanoformulations of phytocannabinoids have been published elsewhere [22,34]. Here, we focus on nanodrug delivery systems developed for cannabinoid derivatives and synthetic compounds acting on the cannabinoid system and designed for various therapeutic applications. The publications included in this review were primarily identified through a PubMed database search, which was conducted using combinations of keywords related to cannabinoid ligands (e.g., cannabinoid*, CB13, rimonabant), cannabinoid receptors (e.g., CB1, CB2), mechanisms of action (e.g., agonist*, antagonist*), structural features (e.g., synthetic, derivative*), and formulation strategies (e.g., nano*, liposom*, micell*). Additionally, a snowballing strategy was applied, and relevant articles were retrieved from the reference lists of the initially selected publications. An overview of formulations for synthetic and chemically modified cannabinoids developed in the reviewed studies is presented in Table 1.

**Figure 4:**
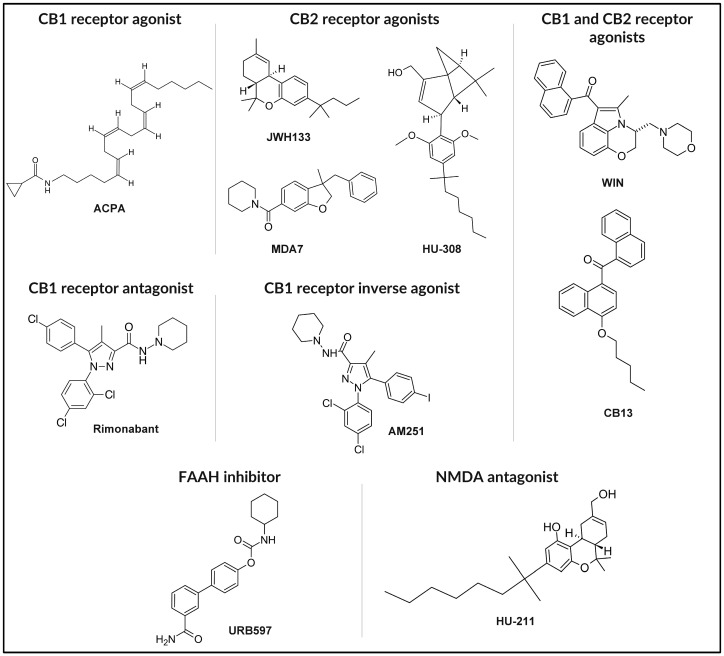
Chemical structures of representative synthetic cannabinoids and endocannabinoid modulators.

The chemical structures of the synthetic and chemically modified cannabinoids discussed in this section, including ACPA, MDA7, HU-308, Rimonabant, AM251, CB13, URB597, and HU-211, are presented in Figure 4.

### 3.1. Δ^9^-Tetrahydrocannabinol-Valine-Hemisuccinate

Given that dicarboxylic acids exist in an ionized form at physiological pH, they enhance the solubility of a substance with which they form an ester. With this in mind, Hingorani et al. [90] synthesized Δ^9^-tetrahydrocannabinol hemisuccinate, a prodrug designed to increase the solubility and permeability of THC. However, the synthesized compound exhibited suboptimal water solubility despite showing improvement compared to the parent molecule. Furthermore, its physicochemical properties, being sticky and resinous, posed challenges in its formulation. In addition to this, Δ^9^-tetrahydrocannabinol hemisuccinate proved unstable and susceptible to oxidation [91]. To improve its stability, researchers then formulated the Δ^9^-tetrahydrocannabinol-amino acid (valine)-dicarboxylic acid (hemisuccinate) ester (THC-VHS). Earlier research had revealed that combining the cannabinoid with the amino acid alone ensured the stability of the compound; however, it did not provide sufficient solubility at physiological pH. Therefore, to ensure both solubility and stability, they decided to combine THC, valine, and hemisuccinate. The resulting THC-VHS showed a 96-fold increase in solubility compared to THC and demonstrated enhanced in vitro permeability [92].

Taskar et al. [66] developed SLNs containing THC-VHS to increase the bioavailability of THC after ocular administration for the treatment of glaucoma. THC exhibits therapeutic effects in this condition by enhancing the outflow of aqueous humor, opening the endothelial-lined Schlemm’s channels, and causing vasodilation through CB1 receptor activation. It also acts as an antioxidant and scavenges free radicals to protect neurons, while its inhibition of glutamic acid release prevents apoptotic ganglion cell death. THC reduces intraocular pressure in marijuana smokers; however, the side effects accompanying this action make systemic administration of THC unsuitable for glaucoma treatment, necessitating the invention of a carrier that can effectively deliver THC locally. Previously tested oil formulations did not provide sufficient drug bioavailability. In addition to SLNs with the prodrug, the researchers also synthesized SLNs containing THC alone for comparison to determine the action of its synthetic derivative and an NE containing only THC-VHS to assess the impact of the formulation on the obtained results. A study conducted on a rabbit model confirmed that the use of nanoformulations could ensure the action of cannabinoids in the eye after local administration. When administered in the form of SLNs, the prodrug reduced intraocular pressure for longer than the emulsion. Particles containing THC and its prodrug, when administered in multiple doses, demonstrated a more potent and long-lasting effect than currently used glaucoma medications, such as pilocarpine hydrochloride or timolol maleate [66].

Sweeney et al. [67] developed a THC-VHS-containing nanoemulsion optimized for duration of activity and drug loading. They investigated the stability of the emulsion at 4 °C and the effect of the sterilization process and surfactant concentration on its intraocular pressure-lowering activity in the New Zealand white rabbits model. The developed NE consists of sesame oil, Tween 80, and Poloxamer 407. The researchers modified the active substance and surfactant amount to investigate their impact on the physicochemical properties and the observed pharmacological effect. The optimized formulation selected for further evaluation contained 2% *w*/*v* Tween 80, characterized by a mean particle size between 170 and 180 nm with PDI 0.04 to 0.1. Zeta potential was around −35 to −40 mV. This study demonstrated that the optimized THC-VHS-containing NE exerted a significantly better intraocular pressure reduction profile in the test model and longer duration of activity compared to the commercial ophthalmic solutions evaluated (timolol and latanoprost ophthalmic solutions, as well as Tocrisolve™) [67].

### 3.2. Naphthalen-1-yl-(4-pentyloxynaphthalen-1-yl)methanone

Naphthalen-1-yl-(4-pentyloxynaphthalen-1-yl)methanone (CB13) was developed in response to the need for a pain-relieving drug effective in conditions resistant to conventional xenobiotic-based therapies. It acts as a peripheral, mixed CB1/CB2 receptor agonist. Activation of CB1 and CB2 receptors in peripheral tissues results in analgesic effects, while stimulation of the cannabinoid receptors in the CNS leads to undesired psychotropic effects. Hence, there is a need to develop a drug that selectively stimulates cannabinoid receptors in peripheral tissues without crossing the blood–brain barrier. CB13 exhibits strong activity [33], but due to its lipophilic nature, it is poorly soluble and consequently not fully absorbed in the body [93].

Martín-Banderas et al. [70] developed polymeric nanoparticles containing CB13, characterized them, and investigated their in vitro cytotoxicity and safety for potential use as an oral cannabinoid delivery system. The researchers modified the synthesis conditions of selected particles and investigated how these changes affected the properties of the resulting carriers. They used various PLGA copolymers as matrix materials, identifying the polymer type as the key factor influencing particle size. Notably, particle size decreased with increasing hydrophobicity of the employed copolymer. The smallest particles were obtained using Resomer 504, 504H, and 752S. The surface charge, which had an absolute value greater than 24 mV for the developed formulations, indicated high system stability. The influence of the surfactant amount on particle size was also investigated, showing that too low a concentration (or complete omission of surfactant addition) resulted in an increase in particle diameter to 600 nm and system heterogeneity. However, the amount of cannabinoid incorporated into the nanoformulation did not affect particle size. CB13’s poor solubility contributed to high encapsulation efficiency for all particle variants, and in vitro studies demonstrated a lack of toxicity [70].

The encapsulation of CB13 in PLGA nanoparticles was also described by Durán-Lobato et al. [71]. Their study involved synthesizing polymeric nanoparticles with incorporated cannabinoid and characterizing their properties depending on the substance used for surface modification of the nanoparticles. Vitamin E, chitosan, Eudragit^®^ RS, and lecithin were selected as substances expected to enhance particle absorption when attached to their surface. The obtained spherical nanoparticles, depending on their composition, had sizes ranging from 253 to 344 nm. The type of surface-modifying agent used did not have a statistically significant effect on particle size. Despite substantial differences in the zeta potential of the systems, the absolute values for all modifications indicated high stability. The positive surface charge of chitosan- and Eudragit-modified particles suggests that these substances may be the most suitable for oral administration, as a positive charge promotes increased mucoadhesion at the site of administration. Nanoparticle entrapment efficiency ranged from 71 to 85.5%, and drug loading was between 8 and 11%. The release of the substance from the carrier occurred the fastest for lecithin-containing particles, but ultimately, all versions provided extended cannabinoid release. Studies on cell lines demonstrated that approximately 100% of cells survived 24 h when exposed to nanoparticles in all tested variants and concentrations. After 48 h, a decrease in cell survival was observed for cells incubated with 0.3 and 3 μM of CB13. Biodistribution studies showed that none of the surface modifications of nanoparticles prevented phagocytic uptake of nanoparticles [71].

Berrocoso et al. [72] developed three types of PLGA nanoparticles loaded with CB13 and assessed their physicochemical properties. The formulations included unmodified PLGA nanoparticles, PLGA nanoparticles coated with PEG chains, and PLGA nanoparticles with covalently bound PEG, which enhanced surface hydrophilicity. The third formulation was characterized by sufficient drug loading (13%) and a particle size below 300 nm. The animal nociceptive behavioral studies showed satisfactory analgesic efficacy after a single oral dose administration with a long, sustained pain-relieving effect, lasting up to eleven days. The observed effect was dose-dependent. The CB13-containing PLGA nanoparticles with covalently bound PEG exhibited a delayed analgesic effect of up to 0.5, 3, or 9 h, depending on the CB13 dose administered (6.8, 3.4, or 1.7 mg/kg, respectively) [72].

In addition to polymeric ones, lipid nanoparticles (LNPs) have also been developed as a formulation for CB13. Since surface modification of nanoparticles may improve their adhesion to the oral mucosa and prolong the systemic circulation time of such formulations, Durán-Lobato et al. [73] investigated chitosan- and PEG-modified PLGA and lipid nanoparticles as potential oral delivery systems for CB13. The developed formulations were characterized by negative zeta potential and the particle size of 320–420 nm and 120–160 nm for PLGA and lipid nanoparticles, respectively. The assessment of in vitro uptake of the developed formulations by the Caco-2 cells, which are used to evaluate the gastrointestinal tract permeability, and THP1 cells (human monocytic cell line), was performed. This study showed that surface modification using PEG led to limited uptake by Caco-2 cells compared to the chitosan-modified nanoparticles. The authors also observed differences in cellular uptake depending on the core of the nanoparticles. PLGA nanoparticles showed a slightly decreased uptake by the Caco-2 cells and increased phagocytic uptake compared to lipid nanoparticles, probably due to the larger particle size of these formulations [73].

Durán-Lobato et al. [74] attempted to optimize the synthesis process of LNPs, using the solvent-emulsion evaporation method for their preparation. The factors modified during the synthesis included the homogenization and sonication times, the type of lipid and its ratio to the solvent, and the concentrations of emulsifiers. By adjusting the homogenization and sonication times, depending on the lipid used, particles of 112 or 138 nm in size were obtained, with a polydispersity index of around 0.3 in both cases. Further studies showed that reducing the solvent-to-lipid ratio or the volume of emulsifier used resulted in increased particle sizes. The tested samples exhibited high entrapment efficiency, ranging from 70% to 76%. Adding lecithin further increased this value, even up to 100%. Loading capacity for formulations without and with lecithin was 7% and 10%, respectively. By entrapping the cannabinoid in the nanoformulation, extended release of the substance lasting up to 7 h was achieved. The impact of storage and the conditions in the gastrointestinal tract on the stability of the nanoparticles obtained under optimized conditions was also examined regarding changes in their size and zeta potential. The stability of the carriers was confirmed after storage at 4 °C for 2 months, and it was demonstrated that the addition of lecithin prevented an increase in particle size in an acidic environment. Studies on murine and human cell lines showed no cytotoxicity of LNPs on cells for 24 h [74].

### 3.3. WIN55,212-2

WIN55,212-2 (WIN) is a synthetic cannabinoid with a high affinity for the CB1 receptor and confirmed analgesic effects [94,95]. Its incorporation into MCs consisting of styrene-maleic acid has been described in several published studies. Linsell et al. [75] developed WIN-loaded MCs, intending to investigate their potential as a pain-relieving drug for neuropathic pain. WIN, as an agonist of cannabinoid receptors, exhibits both analgesic and psychotropic effects. Therefore, to limit its action in the CNS, the researchers developed MCs containing the cannabinoid, designed to selectively target inflamed tissue while avoiding penetration into the brain. The study involved synthesizing and characterizing MCs and in vivo tests using a rodent model to evaluate the impact on neuropathic pain and motor impairment.

The MCs were engineered to achieve targeted loading efficiencies of 5%, 10%, and 25%, with a successful synthesis of MCs matching these parameters. The actual loading efficiencies achieved were approximately 5%, 15%, and 27%. Particle sizes obtained were optimal for the study’s goals; the MCs were sufficiently large to avoid penetrating the blood–brain barrier and healthy blood vessels, reducing renal clearance, thus ensuring prolonged circulation in the bloodstream, yet small enough to penetrate tumor tissue via leaky blood vessels. For instance, MCs with a 15% drug loading had an average diameter of 116.2 nm, while those with a 27% loading measured around 101 nm. The release rate of the cannabinoid was the slowest in MCs with 27% loading, particularly at a pH of 5.5.

The rotarod test, which showed that the rodent’s motor impairment was similar after administration of the free cannabinoid and its micellar formulation, unexpectedly revealed that the drug rapidly penetrated the CNS even when enclosed in styrene-maleic acid. However, this does not mean that the MCs and the free cannabinoid had the same effect on the nervous system, as allodynia was attenuated for 8 h longer after administration of the nanoformulation compared to the free drug. The authors explain this result by the possible degradation of the MCs, which may have led to the release of part of the drug after administration into the bloodstream. In contrast, the prolonged release of the remaining cannabinoid in the MCs provided action lasting up to 8 h [75].

Xian et al. [76] also attempted to encapsulate WIN within MCs composed of styrene-maleic acid. They replicated the MC preparation method used by Linsell et al. [75] and similarly characterized the resulting formulation. In their study, however, they evaluated the efficacy of the obtained formulation (compared to free WIN) on cell lines of triple-negative breast cancer, hormone receptor-positive breast cancer, and castration-resistant prostate cancer. They obtained similar results to the previous study—synthesizing MCs with an average diameter of approximately 132.7 nm, loading efficiencies of 15.3 and 15.5% for two batches, and a prolonged release at pH 5.5 compared to pH 7.4 [76].

Examination of cell lines showed that in both free and MC-encapsulated forms, WIN induced a dose-dependent cytotoxic effect on all tested cancer cells. It was found that although the MC form may limit the occurrence of adverse effects, it was as effective as the free drug in cancer treatment [76]. Greish et al. [77] published another study describing the investigation of the anticancer activity of MCs constructed from styrene-maleic acid with incorporated WIN (SMA-WIN). Similar to their predecessors, they utilized the same method for MC synthesis but went a step further in their research on the anticancer activity of the cannabinoid, examining the therapeutic effect of SMA-WIN in an animal model and additionally studying the in vitro and in vivo combination of the obtained formulation with doxorubicin, a drug used in cancer therapy. The obtained SMA-WIN had a drug loading of 18% with an average size of 152 nm (PDI 0.166) [77].

In vivo, distribution analysis revealed a significant difference in the presence of the cannabinoid in specific tissues, including the tumor, after administration of WIN in MCs compared to the free drug. Injection of the nanoformulation provided a several-fold increase in drug content. On the other hand, 30 min after administration, the MC concentration in the brain was lower than that of free WIN, suggesting reduced psychoactive effects of the MCs. A 12-day study in mice demonstrated a dose-dependent reduction in tumor growth by WIN in MCs compared to the free drug and the control (no significant difference was detected between the free cannabinoid and the control). The drug was found to penetrate the brain, as assessed by measuring mouse mobility, which was reduced for an average of 47 min upon WIN administration, whereas administering its micellar form shortened this time to a minimum of 1.8 min. It is worth noting that this effect was dose-dependent [76].

The cytotoxicity test of the cannabinoid combined with doxorubicin revealed that neither WIN nor WIN in MCs affected cell survival, nor did their combination with doxorubicin at a concentration of 0.3 µM. However, doxorubicin at a concentration of 1 µM reduced survival by approximately 10%, and its combination with WIN and WIN encapsulated in a nanoformulation resulted in a greater reduction of this parameter by 86% and 73%, respectively. Finally, the effect of combining doxorubicin with MCs in vivo was investigated, and the results demonstrated a stronger tumor-reducing action for the combination compared to doxorubicin and MCs administered separately [76].

### 3.4. JWH133

JWH133 is a synthetic agonist devoid of psychoactive effects, with a 200-fold greater selectivity for the CB2 receptor over CB1. JWH133 exhibits a broad spectrum of pharmacological activity. It exerts anticancer, cardioprotective, hepatoprotective, gastroprotective, nephroprotective, antihyperalgesic, and immunomodulatory activities. This substance also protects against oxidative damage and inflammation, inhibits fibrosis and apoptosis, and acts as an immunosuppressant [96]. Due to the pleiotropic effect of JWH133, there is an effort to obtain formulations that allow its delivery to the site of action.

Qiu et al. [78] developed a nanoformulation for JWH133 to enhance its absorption and half-life while simultaneously reducing its side effects. The formulation was designed for potential use in the treatment of rheumatoid arthritis. The synthetic cannabinoid was encapsulated in porous, hollow nanoparticles constructed from copper sulfide (CuS). After the drug was incorporated into the particles, they were coated with a hyaluronic acid (HA) layer (CuS-JWH133@HA). This carrier structure was intended to provide a two-stage release of JWH133 at the target site of action. JWH133 was efficiently incorporated into the mesoporous copper sulfide skeleton. Modifying the surface of the CuS-JWH133 using HA aimed to prevent the untimely leakage of the loaded drug. The developed formulation showed a satisfactory effect in vitro and in vivo rheumatoid arthritis models, proving the therapeutic potential of JWH133 in this condition [78].

### 3.5. Rimonabant

Rimonabant is a CB1 receptor antagonist. It was found to effectively reduce body weight in obese and overweight individuals and ameliorate metabolic abnormalities related to these conditions, including hepatic steatosis, insulin resistance, and type 2 diabetes. However, due to its neuropsychiatric side effects, including depression, anxiety, and suicidal ideation, this medicine was withdrawn from the pharmaceutical market in 2009, and its clinical application was halted [97,98,99,100,101].

Hirsch et al. [79] developed novel rimonabant-loaded PLGA NPs to increase hepatic targeting and limit the side effects. The obtained NPs were characterized by a diameter of ~250 nm, a relatively narrow PDI, and a negative zeta potential. The biodistribution study showed that the formulation accumulated primarily in the liver, followed by the kidney, intestine, spleen, and fat, while only a small amount was detected in the lungs. It was proven that only a negligible amount reached the brain, which should be sufficient to limit neuropsychiatric side effects. The in vivo studies of the developed formulation showed no CNS-mediated behavioral activities in contrast to treatment with free rimonabant. Chronic treatment of diet-induced obese mice with rimonabant-loaded PLGA NPs led to the reduction of hepatic steatosis and liver injury, along with enhancement of insulin sensitivity. The authors showed that an appropriate delivery system allowing for hepatic targeting may restore the metabolic advantages of this synthetic cannabinoid in peripheral tissues, limiting the negative side effects [79].

Esposito et al. [80] addressed the challenge of developing cannabinoid-loaded NLCs. They used rimonabant as a prototypical cannabinoid antagonist and successfully incorporated it into the lipid core of the developed carriers using two alternative methods, i.e., adding the lipid phase into the aqueous one (direct protocol) or the aqueous phase into the lipid one (reverse protocol). The developed formulations were similar in morphology but differed in entrapment efficiency. The reverse protocol allowed investigators to achieve 98% entrapment efficiency compared to 67% achieved with the direct protocol. The NLCs obtained using the second method were treated with polysorbate 80 and then subjected to the in vivo examination after intranasal administration to rats. The biodistribution study showed increased rimonabant concentration in the brain compared to the drug in solution. The authors concluded that the developed delivery system was an optimal strategy that improved the low solubility of cannabinoid compounds in an aqueous system and was suitable for in vivo administration [80].

### 3.6. URB597

URB597 is a potent and selective fatty-acid amide hydrolase (FAAH) inhibitor that elicits an analgesic effect. In contrast to rimonabant, it does not cause any undesirable side effects associated with cannabinoid receptor activation. Its mechanism of action is mediated by increasing the concentration of endocannabinoids, such as AEA and 2-AG. These endogenous cannabinoids are potent neurotransmitters that relieve inflammatory and neuropathic nerve injury-induced pain. FAAH is one of the enzymes that degrade endocannabinoids. Therefore, the administration of URB597, an FAAH inhibitor, prolongs their action [102].

Esposito et al. [81] proposed a reverse method of preparing NLCs, which successfully incorporated URB597 into such a delivery system. The studied approach helped solve the problem of poor recovery of encapsulated drugs and consisted of pouring the water phase, which contained a 2.5% (*w*/*w*) aqueous poloxamer 188 solution, into the oil phase composed of a lipid mixture of tristearin/miglyol 2:1 (*w*/*w*). The URB597-containing NLCs were characterized by a Z-average of 242.4 ± 4.5 nm and a satisfactory encapsulation efficiency of 92.8 ± 0.8%. The authors did not perform any in vitro or in vivo studies nor indicate the route of administration of such a formulation. However, they concluded that the developed NLCs were suitable for in vitro and in vivo clinical and preclinical studies [81].

### 3.7. AM251

The same work also addressed the problem of AM251 encapsulation into NLCs. AM251 is an inverse agonist of the cannabinoid receptor CB1 [81]. There is considerable evidence that the endocannabinoid system plays a significant role in treating disorders that might have a component of excess appetitive drive and conditions associated with it [103]. A low dose of AM251 is capable of producing significant changes in energy balance, leading to reductions in food intake and body weight in rats treated either daily or infrequently. Chambers et al. [104] showed that this reduction is independent of side effects and lasted longer than the pharmacological blockade of CB1 receptors in the brain. Moreover, AM251 regulates the expression of the epidermal growth factor receptor and its ligands via destabilization of nuclear estrogen-related receptor α protein and, therefore, may exert anticancer action [105]. AM251 is a presumed selective CB1 receptor antagonist. However, Seely et al. [106] showed that this agent binds with mid-nanomolar affinity to human mu-opioid receptors. It was also proven to competitively antagonize morphine-induced G-protein activation in Chinese hamster ovary cells stably expressing human mu-opioid receptors. AM251 not only blocks morphine inhibition of cAMP production but also elicits cAMP rebound in cells chronically exposed to morphine [106]. Esposito et al. [81] successfully incorporated AM251 into NLCs with 99.9 ± 0.1% encapsulation efficiency. The developed nanocarriers were characterized by a Z-average of 231.0 ± 3.3 nm. Unfortunately, this study did not attempt to compare the biological effect of the developed formulation with the pure substance, nor did it include in vitro or in vivo studies. The authors focused on the comparison of two methods of preparing NLCs and indicated that the indirect method appeared superior to the direct one [81].

### 3.8. MDA7

CB2 receptor agonists are being sought for use in the treatment of different types of pain. MDA7 is known as a selective agonist of CB2 receptors [107]. Naguib et al. [107] investigated the effect of MDA7 in reversing neuropathic pain in spinal nerve ligation and paclitaxel-induced neuropathy models in rats, showing that this agent attenuated tactile allodynia in a dose-related manner. It was also proven that the reduction of neuropathic pain was not associated with any effect on locomotor behavior, which is a good prognosis for the further development of this compound in therapy [107]. The effect of MDA7 in paclitaxel-induced neuropathy is associated with the modulation of genes encoding glutamate transporters and N-methyl-D-aspartate receptor 2B, which are molecules important in central sensitization, and neuro-immune-related genes, such as neuronal nitric oxide synthase, chemokine CX3CL, toll-like receptor 2 (TLR2), as well as leptin. In addition to the direct influence on gene expression, MDA7 was shown to act through inflammatory pathways by affecting the signaling of tumor necrosis factor (TNF), nuclear factor kappa-light-chain-enhancer of activated B cells (NF-κB), transforming growth factor beta (TGFβ), and mitogen-activated protein kinases (MAPK) [108], and by suppressing the overexpression of brain-derived neurotrophic factor in microglia [109]. Xu et al. [110] investigated the role of MDA7 in the treatment of chronic post-ischemia pain in rats used as a model of complex regional pain syndrome type 1. The authors proved that intraperitoneal administration of MDA7 attenuated mechanical allodynia by suppressing peripheral edema and inhibiting spinal microglial activation as a result of reduced CX3CR1 and CB2 receptor expression. Moreover, the authors showed that MDA7 mitigated the loss of intraepidermal nerve fibers induced by chronic post-ischemia pain [110].

Astruc-Diaz et al. [83] compared the antiallodynic effect of different types of vehicles for MDA7 after intravenous administration in rats. For this purpose, the investigators developed MDA7-loaded hydroxypropyl-β-cyclodextrins (HPβCD), a micellar preparation, and liposomes. The morphology and size of the obtained structures were evaluated by dynamic light scattering and transmission electron microscopy. HPβCD was demonstrated to successfully deliver chiral drugs, such as MDA7, since such a formulation ensured an equal proportion of each enantiomer. The HPβCD-based and micellar formulations improved the antiallodynic effect of MDA7 in comparison to their liposome-based counterpart. The authors concluded that several factors may be responsible for the observed results, including the hydrophobic-core–hydrophilic-shell structure of cyclodextrin- and MC-based systems, and their smaller sizes compared to liposomes. These features could prevent the system’s recognition by the mononuclear phagocyte system, leading to prolonged circulation of the drug. The authors also pointed out that a micellar system may be a suitable strategy when a lipophilic compound has inappropriate geometry, size, or physicochemical properties to form a host–guest complex characteristic of an HPβCD-based system [83].

### 3.9. Arachidonoylcyclopropylamide

Cannabinoid ligands have been proven to exert antiproliferative effects and induce apoptosis in numerous cancers. Boyacıoğlu et al. [84] showed that the synthetic CB1 receptor-specific ligand arachidonoylcyclopropylamide (ACPA) presents a pro-apoptotic effect in non-small cell lung cancer. The biochemical mechanism of this phenomenon is associated with the inhibition of the Akt/PI3K pathway, glycolysis, the tricarboxylic acid cycle, amino acid biosynthesis, the urea cycle, and the activation of the JNK pathway. Nevertheless, an appropriate delivery system for this active substance was sought due to its poor chemical stability within 24 h. The developed ACPA-loaded PCL-NPs improved the stability and prolonged the action of ACPA in vitro. ACPA-loaded PCL-NPs were characterized by a mean particle size, PDI, and zeta potential of 162.2 ± 2.3 nm, 0.251 ± 0.008, and −29.4 ± 0.6 mV, respectively. The authors achieved a satisfactory ACPA loading of 39.9 ± 14.7% and a sustained release from the NPs of 63.9% over 7 days [84].

### 3.10. HU-308

HU-308 is a cannabinoid derivative known to be a cannabinoid agonist with strong selectivity for the CB2 receptor, hundreds of times higher than for the CB1 receptor [111]. Many researchers have demonstrated the pleiotropic effects of this compound. HU-308 can stimulate osteoblast proliferation and activity [112], mediate anti-inflammatory effects in synovial fibroblasts and macrophages following joint injury [113], and reduce corneal hyperalgesia and inflammation [114]. Te Boekhorst et al. [85] developed di-oleoyl-PEG-phosphatidylethanolamine 1000- and di-stearoyl-PEG-phosphatidylethanolamine 2000-based paramagnetic MCs loaded with HU-308 to target the CB2 receptor present in the atherosclerotic plaque macrophages. In vitro and in vivo studies showed their potential as magnetic resonance contrast agents for the visualization of vulnerable plaque [85].

### 3.11. HU-211

11-hydroxy-∆8-tetra-hydrocannabinol dimethylheptyl (HU-211) is a synthetic cannabinoid derivative lacking psychotropic activity and known to exert an ocular hypotensive effect [86]. Naveh et al. [86] developed a medium-chain triglyceride-based HU-211-loaded oil-in-water submicron emulsion characterized by a mean droplet size of 100 ± 20 nm and a mean zeta potential of −31 mV. The authors investigated the effect of the HU-211-loaded emulsion on intraocular pressure in normotensive rabbits, showing that this synthetic cannabinoid, which is almost insoluble in water, can penetrate the eye when incorporated into a developed delivery system and exert a significant ocular hypotensive effect lasting over 6 h [86].

HU-211 also demonstrated neuro- and cerebral-protective effects due to its anti-inflammatory and antioxidant activities [115], mediated by inhibiting NF-κB and decreasing cytokines, such as TNFα and interleukin-6, which could ensure the integrity of the blood–brain barrier and reduce cell apoptosis and death [116,117]. Shohami et al. [118] and Maas et al. [119] investigated its efficacy in closed-head injury and severe traumatic brain injury in animals and humans, respectively. The in vivo studies on humans and rats showed the safety and efficacy of HU-211 in the tested models [118,119]. Since curcumin has been found to have antidepressant effects in major depression models, its combination with HU-211, which exerts a significant role in brain disease research as a novel therapeutic tool for depression treatment, seems an interesting approach. He X et al. [87,88,89] developed curcumin- and HU-211-loaded SLNs and investigated their antidepressant effect in different models. An emulsification and low-temperature solidification method allowed for the development of SLNs with the loading efficiencies of curcumin and HU-211 of about 19% and 0.80%, respectively. The developed delivery system was characterized by a spherical shape, uniform size, and a negative zeta potential. Treatment with curcumin- and HU-211-loaded SLNs induced greater dopamine/5-hydroxytryptamine release with reduced corticosterone-induced apoptotic cell death in vitro. Additionally, the in vivo studies showed that such a formulation positively affects the recovery from depressive behavior by increasing the levels of dopamine/5-hydroxytryptamine and the expression of CB1, p-MEK1, and p-ERK1/2 in the hippocampus and striatum [87]. Moreover, as shown in another study, curcumin- and HU-211-loaded SLNs reduced the immobility time in the forced swim test, enhanced fall latency in the rotarod test, and improved the dopamine levels in mouse blood [88]. Finally, the antidepressant effects of the developed formulation were investigated in wild-type (CBR1+/+) and cannabinoid receptor 1 knockout (CBR1−/−) mouse models of corticosterone-induced major depressive disorder. Intraperitoneal administration of curcumin (20 mg/kg) and HU-211 (0.85 mg/kg) in SLNs for two successive days promoted the release of dopamine and norepinephrine and simultaneously improved rat locomotor function in a wild-type mouse model. Interestingly, the absence of antidepressant effects in the CBR1−/− mouse model of major depressive disorder suggests that CBR1 is necessary for the effects observed in response to the proposed treatment. Nevertheless, the authors concluded that the developed NPs should be further optimized to improve their performance and that their biocompatibility still requires careful investigation [89].

## 4. Discussion

This review highlights the multifaceted strategies developed to enhance the therapeutic potential of synthetic and chemically modified cannabinoids using nanotechnology-based delivery systems. Across the analyzed studies, nanoformulations consistently mitigate the intrinsic limitations of cannabinoids, including low solubility, poor oral bioavailability, and variable pharmacokinetics. Lipid-based nanocarriers, polymeric NPs, MCs, and hybrid systems improve drug stability, enable site-specific delivery, and, in some cases, prolong pharmacological effects while reducing systemic exposure. These results align with broader trends in nanomedicine, where rational carrier design critically influences therapeutic performance [23,34].

Despite these advantages, translation to clinical use remains challenging. Most cannabinoid nanoformulations have not advanced beyond preclinical evaluation due to the absence of harmonized regulatory pathways, the difficulty of ensuring batch-to-batch reproducibility, and the limited availability of standardized immunogenicity and toxicity assays [120,121]. Furthermore, hybrid nanocarriers often fall outside conventional small molecule or biologic frameworks, as illustrated by the limited number of FDA-approved protein nanoparticles, whose approval has been constrained by production variability and immunogenic risks [122]. To unlock the full therapeutic potential of nanoformulated cannabinoids, harmonized international guidelines, validated preclinical to clinical bridging studies, and standardized toxicity testing will be essential [123].

Advances in nanotechnology provide a foundation for next-generation cannabinoid therapeutics with enhanced efficacy and safety. Future nanoformulations are likely to incorporate multifunctional and intelligent features, such as stimulus-responsive release, co-delivery of synergistic agents, and real-time imaging for biodistribution tracking. Hybrid lipid polymer systems, dendrimers, and bioinspired nanocarriers offer opportunities to improve tissue specificity, cross biological barriers, and reduce systemic side effects.

Integration with precision medicine will be key, with biomarker-guided patient stratification helping to optimize therapeutic outcomes. Moreover, AI-assisted design and digital twin simulations could accelerate the development of nanocarriers with predictable pharmacokinetics and scalable manufacturing, streamlining regulatory approval.

## 5. Conclusions

Nanotechnology-enabled delivery of synthetic and chemically modified cannabinoids offers a promising approach to overcome the intrinsic limitations of these compounds, positioning them as candidates for future precision therapeutics. Preclinical evidence supports their enhanced stability, bioavailability, and site-specific activity; however, translating these findings into clinical applications will require coordinated advances in innovation, manufacturing, and regulatory science. Nanoformulated cannabinoids have the potential to transform therapeutic strategies, but their clinical impact will depend on harmonized regulations, standardized testing, and robust translational research. With sustained international collaboration, these next-generation formulations may become a key component of personalized medicine in the coming decade.

## Figures and Tables

**Table 1:** Overview of nanoformulations for synthetic and chemically modified cannabinoids.

Cannabinoid Type	Compound Name and Structure	Delivery System	Key Findings	Ref.
Prodrug of THC	Δ^9^-Tetrahydrocannabinol-valine-hemisuccinate (THC-VHS)	SLNs, NE	THC-VHS-loaded SLNs more effectively and for longer reduced intraocular pressure in rabbits vs. NE and standard treatments; increased drug levels in eye tissues.	[66]
		NE	THC-VHS-loaded NE reduced intraocular pressure more effectively and for longer vs. timolol, latanoprost, and other tested formulations.	[67]
	Hemiglutarate ester prodrug of THC (THC-HG)	MCs	MCs enhanced THC-HG delivery to the anterior ocular chamber, but further research is needed to improve penetration to the posterior eye tissues.	[68]
CBD analogue	CBD–monovalinate–monohemisuccinate (CBD-Mono-VHS), CBD-divalinate-dihemisuccinate (CBD-Di-VHS)	NE	CBD analogs penetrated ocular tissues better than CBD; CBD-Di-VHS and CBD-Mono-VHS showed improved permeation, likely due to enhanced stability and optimized physicochemical properties.	[69]
CB1 and CB2 peripheral receptor agonists	1-Naphthalenyl [4-(pentyloxy)-1-naphthalenyl]methanone) (CB13)	PLGA NPs	More hydrophobic polymers led to smaller size; smaller particles released drug faster; polymers with lower molecular weight and lactide content increased water absorption and erosion; non-cytotoxic.	[70]
			Lecithin and vitamin E modifications increased release rate; chitosan and Eudragit RS enhanced mucoadhesion; chitosan-PLGA showed highest Caco-2 uptake; accumulation mainly in liver and spleen.	[71]
		PLGA-PEG NPs	Animal pain-behavior studies, using paw pressure and acetone tests, demonstrated that compared to free CB13, CB13-PLGA-PEG NPs provided significantly enhanced analgesic effects, sustaining pain relief for up to eleven days after a single oral dose.	[72]
		PLGA-PEG NPs, PLGA-PEG-CS	Formulations were blood-compatible and non-cytotoxic to Caco-2 cells; CS coating increased interaction with Caco-2 cells and limited uptake by THP-1 cells; PEG coating reduced uptake by Caco-2 cells and prevented uptake by THP-1 cells.	[73]
		LNPs	CB13-loaded LNPs showed high encapsulation efficiency and drug loading when lecithin was included; stable in simulated intestinal conditions; non-cytotoxic to NIH 3T3 and HEK 293T cells.	[74]
	R-(+)-[2,3-Dihydro-5-methyl-3-(4-morpholinylmethyl)pyrrolo [1,2,3-de]-1,4-benzoxazin-6-yl]-1-naphthalenylmethanone (WIN)	SMA	SMA-WIN provided longer-lasting neuropathic pain relief than standard WIN (up to 8 h); in the rotarod test, motor impairment resolved faster than with WIN; may reduce CNS-related side effects.	[75]
			SMA-WIN showed cytotoxic effects comparable to free WIN in triple-negative breast cancer, hormone receptor-positive breast cancer, and castration-resistant prostate cancer cell lines.	[76]
			SMA-WIN showed strong cytotoxicity against triple-negative breast cancer cells; in animal models, markedly reduced tumor growth compared to free WIN; low doses of SMA-WIN combined with doxorubicin enhanced anticancer effect without increasing toxicity.	[77]
CB2 receptor agonist	(6AR,10AR)-3-(1,1-Dimethylbutyl)-6A,7,10,10A-tetrahydro-6,6,9-trimethyl-6H-dibenzo[B,D]pyran (JWH133)	Mesoporous copper sulfide (CuS) with HA cover	JWH133 targeted CB2 receptors on macrophages, synovial cells, and osteoblasts; inhibited inflammatory factor secretion and enhanced osteoblast activity; CuS-JWH133@HA reduced inflammation levels in vivo and improved condition of inflamed and swollen joints in mice.	[78]
CB1 receptor antagonist	5-(4-Chlorophenyl)-1-(2,4-dichlorophenyl)-4-methyl-N-(piperidin-1-yl)-1H-pyrazole-3-carboxamide (rimonabant)	PLGA NPs	PLGA nanoparticles enabled liver-targeted delivery of rimonabant, reducing liver fat and improving metabolic parameters in animals while avoiding significant central nervous system side effects.	[79]
		NLCs	In vivo, intranasal rimonabant-NLC increased brain drug concentration compared to conventional delivery; enabled targeted CNS delivery with reduced peripheral side effects.	[80]
Fatty acid amide hydrolase inhibitor	(3′-(aminocarbonyl)[1,1′-biphenyl]-3-yl)-cyclohexylcarbamate (URB597)	NLCs	NLCs showed consistent size and stability with high encapsulation efficiency for URB597, AM251, and rimonabant; effective for delivering high cannabinoid concentrations, supporting clinical potential.	[81]
Inverse agonist of CB1 receptor	1-(2,4-dichlorophenyl)-5-(4-iodophenyl)-4-methyl-N-(piperidin-1-yl)-1H-pyrazole-3-carboxamide (AM251)			
Inverse agonist of CB1 receptor	5-(4-Chlorophenyl)-1-(2,4-dichlorophenyl)-4-methyl-N-(piperidin-1-yl)-1H-pyrazole-3-carboxamide (rimonabant)			
	(3′-(aminocarbonyl)[1,1′-biphenyl]-3-yl)-cyclohexylcarbamate (URB597)	SLNs, NLCs	SLNs and NLCs enhanced URB597 solubility and brain delivery; polysorbate 80-modified SLNs improved biodistribution and extended circulation time; intranasal URB597-loaded SLNs promoted prosocial behavior in animals.	[82]
CB2 receptor agonist	(1-[(3-benzyl-3-methyl-2,3-dihydro-1-benzofuran-6-yl)carbonyl]piperidine) (MDA7)	HPβCD, MCs, and liposomes	In a rat model of neuropathic pain, HPβCD-MDA7 provided the greatest pain relief compared to MCs and liposomes; improved solubility and reduced immune recognition of HPβCD support its potential for pain management.	[83]
CB1 receptor agonist	N-(Cyclopropyl)-5Z,8Z,11Z,14Z-eicosatetraenamide (ACPA)	PCL NPs	ACPA-loaded PCL NPs improved stability, bioavailability, and sustained release; inhibited cancer cell growth and promoted apoptosis in NSCLC cells; sustained CB1 activation enabled more effective inhibition of the Akt/PI3K pathway and activation of the JNK pathway.	[84]
CB2 receptor agonist	[(1R,4R,5R)-4-[2,6-dimethoxy-4-(2-methyloctan-2-yl)phenyl]-6,6-dimethyl-2-bicyclo [3.1.1]hept-2-enyl]methanol (HU-308)	Paramagnetic MCs	MCs were designed to target CB2 receptors and neutrophil gelatinase-associated lipocalin on macrophages in atherosclerotic plaques; specific binding was confirmed in vitro; in vivo studies confirmed their ability to identify vulnerable plaque areas via MRI and fluorescence microscopy.	[85]
NMDA antagonist	(6aS,10aS)-9-(Hydroxymethyl)-6,6-dimethyl-3-(2-methyloctan-2-yl)-6a,7,10,10a-tetrahydrobenzo[c]chromen-1-ol (HU-211; dexanabinol)	Submicron emulsion	In normotensive rabbits, HU-211, applied as an eye drop formulation, reduced intraocular pressure by 5.3 mmHg (24% of baseline) at 1.5 h post-application, with effects lasting over 6 h; minor intraocular pressure reduction in the untreated eye indicated possible systemic absorption.	[86]
		SLNs	SLNs co-loaded with curcumin and HU-211 increased dopamine and serotonin release and reduced corticosterone-induced apoptosis in vitro; in vivo, improved depressive behaviors in mice, enhanced CB1 expression, and activated MEK1/ERK1/2 pathway; HU-211 SLNs also preserved brain-derived neurotrophic factor and neuronal nuclei expression.	[87]
			Curcumin/SLNs-HU-211 enhanced PC12 cell viability and reduced immobility time in mice with depressive symptoms; activated MEK1/ERK1/2 pathways and CB1 receptors, reducing apoptosis and improving neuronal function.	[88]
			Curcumin/SLNs-HU-211 increased dopamine and norepinephrine expression and activated MEK1/ERK1/2 pathways in CBR1^+/+^ mice, indicating antidepressant and neuroprotective effects; no effects were observed in CBR1^−/−^ mice, confirming CB1 receptor dependency.	[89]

Solid lipid nanoparticles (SLNs); nanoemulsion (NE); micelles (MCs); poly(lactic-co-glycolic acid) (PLGA); nanoparticles (NPs); polyethylene glycol (PEG); chitosan (CS); lipid nanoparticles (LNPs); styrene maleic acid-based nanomicelles (SMA); hyaluronic acid (HA); nanostructured lipid carriers (NLCs); Hydroxypropyl-β-cyclodextrins (HPβCD); Polycaprolactone (PCL).
